# Multimodal machine learning to predict surgical site infection with healthcare workload impact assessment

**DOI:** 10.1038/s41746-024-01419-8

**Published:** 2025-02-23

**Authors:** Kenneth A. McLean, Alessandro Sgrò, Leo R. Brown, Louis F. Buijs, Katie E. Mountain, Catherine A. Shaw, Thomas M. Drake, Riinu Pius, Stephen R. Knight, Cameron J. Fairfield, Richard J. E. Skipworth, Sotirios A. Tsaftaris, Stephen J. Wigmore, Mark A. Potter, Matt-Mouley Bouamrane, Ewen M. Harrison, TWIST Collaborators, K. Baweja, W. A. Cambridge, V. Chauhan, K. Czyzykowska, M. Edirisooriya, A. Forsyth, B. Fox, J. Fretwell, C. Gent, A. Gherman, L. Green, J. Grewar, S. Heelan, D. Henshall, C. Iiuoma, S. Jayasangaran, C. Johnston, E. Kennedy, D. Kremel, J. Kung, J. Kwong, C. Leavy, J. Liu, S. Mackay, A. MacNamara, S. Mowitt, E. Musenga, N. Ng, Z. H. Ng, S. O’Neill, M. Ramage, J. Reed, A. Riad, C. Scott, V. Sehgal, A. Sgrò, L. Steven, B. Stutchfield, S. Tominey, W. Wilson, M. Wojtowicz, J. Yang

**Affiliations:** 1 Department of Clinical Surgery, University of Edinburgh, 51 Little France Crescent, Edinburgh, EH16 4SA UK; 2 Centre for Medical Informatics, Usher Institute, University of Edinburgh, 9 Little France Rd, Edinburgh, EH16 4UX UK; 3 Colorectal Unit, Western General Hospital, Edinburgh, EH4 2XU UK; 4 AI Hub for Causality in Healthcare AI with Real Data, University of Edinburgh, Edinburgh, EH9 3FG UK; 5 Edinburgh Medical School, University of Edinburgh, Edinburgh, UK

**Keywords:** Infectious diseases, Skin manifestations, Prognosis, Health services

## Abstract

Remote monitoring is essential for healthcare digital transformation, however, this poses greater burdens on healthcare providers to review and respond as the data collected expands. This study developed a multimodal neural network to automate assessments of patient-generated data from remote postoperative wound monitoring. Two interventional studies including adult gastrointestinal surgery patients collected wound images and patient-reported outcome measures (PROMs) for 30-days postoperatively. Neural networks for PROMs and images were combined to predict surgical site infection (SSI) diagnosis within 48 h. The multimodal neural network model to predict confirmed SSI within 48 h remained comparable to clinician triage (0.762 [0.690–0.835] vs 0.777 [0.721–0.832]), with an excellent performance on external validation. Simulated usage indicated an 80% reduction in staff time (51.5 to 9.1 h) without compromising diagnostic accuracy. This multimodal approach can effectively support remote monitoring, alleviating provider burden while ensuring high-quality postoperative care.

## Introduction

There has been growing recognition in recent years from governments and healthcare organisations that digital transformation is not just desirable, but essential to the delivery of healthcare in the future^1–3^. These represent potentially large-scale and cost-effective methods to promote healthier lifestyles across populations, and to monitor and manage health conditions^2,4,5^, with emerging real-world case studies^6^. Furthermore, if effectively and equitably implemented within global health systems, these digital health interventions (DHIs) have the potential to enhance the accessibility and efficiency of healthcare^2^. Within the field of surgery, there is accelerating interest in DHIs for remote postoperative monitoring^7^. This is viewed as a route to facilitate rapid recognition and appropriate response to suboptimal patient recovery or potential postoperative complications, both of which are critical in minimising avoidable morbidity and mortality^8^. Such interventions are particularly valuable with the trend towards earlier discharge to the community, as complications that would otherwise have occurred under the direct care of surgical teams will now occur at home^9^.

However, there is widespread acknowledgement that the potential of DHIs has yet to be realised within healthcare systems^10^. As the amount and complexity of data collected on patients expand as part of remote monitoring, this poses even greater burdens on health services to review and respond^11^. Without appropriate staff allocated and/or decision assistance, it is uncertain how these DHIs could be could be effectively integrated into routine clinical practice. The nature of DHIs provide the opportunity to incorporate automated assessment into these remote monitoring pathways. There is early evidence that neural network models can utilise multimodal data to potentially to meet or exceed clinician diagnostic capabilities^12^, although further research is needed to validate these findings in clinical settings. These are a form of deep learning which have proven adept classification by learning hierarchical patterns in the data through multiple layers of nonlinear transformations, enabling the network to identify complex relationships and features^13^. This would facilitate real-time clinical recommendations on a large scale, without significantly burdening healthcare staff. However, there is currently no evidence of the use of these models for automated assessment within remote monitoring pathways in surgery.

Surgical-site infections (SSI) are a promising initial target as one of the most common postoperative complications^14,15^, and being associated with a substantial impact on morbidity and mortality^15,16^, and healthcare utilisation of patients^17^. SSI surveillance through remote wound monitoring has already been shown to allow earlier diagnosis, reduce unnecessary healthcare attendance, and improve the quality of care^18,19^. The symptoms of SSI can be difficult to distinguish from the expected inflammation from the surgical incision, with clinicians needing to consider a combination of patient-reported symptoms, and visual or tactile evidence to diagnose^20^. This can include localised pain, swelling (oedema), redness (erythema), heat (calor), or pus; as well as systemic responses to infection (fever). This multimodal assessment is considered essential for remote postoperative wound surveillance by patients and clinicians^21^, particularly given there is evidence that use of PROMs alone can lead to higher false positive rates (being sensitive but not necessarily specific to the diagnosis of SSI)^18,22^. Therefore, this study aimed to develop a neural network framework for the automated assessment of multimodal patient-generated data (patient-reported outcome measures [PROMs] and wound images) to predict the need for in-person review according to their risk of SSI. Furthermore, it aimed to evaluate strategies for how automated assessment could be effectively implemented within a remote postoperative wound surveillance pathway^7,19^.

## Results

There were 423 patients who received the intervention across the studies: 52.7%, *n* = 223/423 in the “*Tracking wound infection with smartphone technology*” (TWIST) trial and 47.3%, *n* = 200/423 in the *ImplementatioN of Remote Surgical wOund Assessment during the coviD-19 pandEmic*” (INROADE) study. While there were similarities in patient characteristics across both studies (Table 1), there were notable differences which was in part due to the inclusion of patients undergoing elective surgery in INROADE. Those who were enrolled in INROADE were significantly older (mean age: 48.0 vs 41.8, *p* < 0.001), and more likely to be undergoing open or more complex procedures when compared to the TWIST cohort. Furthermore, there was a significantly higher rate of 30-day SSIs observed (16.5% vs 9.4%, *p* = 0.042)

**Table 1:** Comparison of derivation and validation cohort datasets

		Datasets		
		Development (*n* = 200)	External validation (*n* = 223)	*p*
Age (years)	Mean (SD)	48.0 (16.3)	41.8 (17.2)	<0.001
Sex	Male	97 (48.5)	106 (47.5)	0.919
	Female	103 (51.5)	117 (52.5)	
Ethnicity	BAME	5 (2.5)	10 (4.5)	0.402
	White	195 (97.5)	213 (95.5)	
BMI (kg/m^2^)	Not obese (<30 kg/m^2^)	133 (66.5)	161 (73.2)	0.166
	Obese (≥30 kg/m^2^)	67 (33.5)	59 (26.8)	
Diabetes mellitus	No	187 (93.5)	213 (95.5)	0.485
	Yes	13 (6.5)	10 (4.5)	
Operative urgency	Elective	85 (42.5)	0 (0.0)	<0.001
	Emergency	115 (57.5)	223 (100.0)	
Operative approach	Minimally-invasive	119 (59.5)	169 (75.8)	<0.001
	Open	81 (40.5)	54 (24.2)	
Operative contamination	Clean-Contaminated	165 (82.5)	170 (76.2)	0.143
	Contaminated/Dirty	35 (17.5)	53 (23.8)	
Operative complexity	Minor/Intermediate	17 (8.5)	37 (16.6)	<0.001
	Major	159 (79.5)	183 (82.1)	
	Complex Major	24 (12.0)	3 (1.3)	
30-day SSI	No	167 (83.5)	202 (90.6)	0.042
	Yes	33 (16.5)	21 (9.4)	
Time-to-diagnosis of SSI (days)	Mean (SD)	11.3 (5.4)	9.3 (6.3)	0.227
Usage of intervention	No	34 (17.0)	70 (31.4)	0.001
	Yes	166 (83.0)	153 (68.6)	
Number of responses (n)	Mean (SD)	7.0 (3.4)	2.4 (0.9)	<0.001

*BMI* Body mass index, *BAME* Black, Asian, or Minority ethnic, *SD* Standard deviation, *SSI* Surgical-site infection.

Of all patients, 75.4% (*n* = 319/423) submitted a response for clinical review across both studies (Table 1), with a median of 3.0 responses per active patient in TWIST (IQR: 2.0–3.0), and 8.0 in INROADE (IQR: 4.0–10.0). Furthermore, 3.7% (*n* = 57/1545) of responses were submitted within 48 h of a clinical diagnosis of SSI.

Overall, there were 1545 responses containing PROMs collected across the clinical studies. There was significant heterogeneity in symptoms of SSI reported by patients across the studies (Supplementary Fig. 1), with the majority of patients reporting no symptoms (71.9%, *n* = 1111/1545) or isolated symptoms (16.8%, *n* = 260/1545). While the event rate of confirmed SSI within 48 h was low across both studies (INROADE: 3.8% [*n* = 44/1167], TWIST: 3.4% [*n* = 13/378]), the rate of suspected SSI based on remote review of PROMs was higher (INROADE: 14.4% [*n* = 168/1167], TWIST: 15.6% [*n* = 59/378]).

Symptoms of concern highlighted during remote review of PROMs was generally consistent with those observed in patients with confirmed diagnosis of SSI (Supplementary Fig. 1; Supplementary Tables 1-2), although was consistently more conservative. Clinician suspicion of SSI based on remote review of PROMs was associated with an increasing rate of confirmed SSI diagnoses within 48 h from 1.4% (low risk on remote review) to 24.0% (high risk on remote review).

**Fig. 1:**
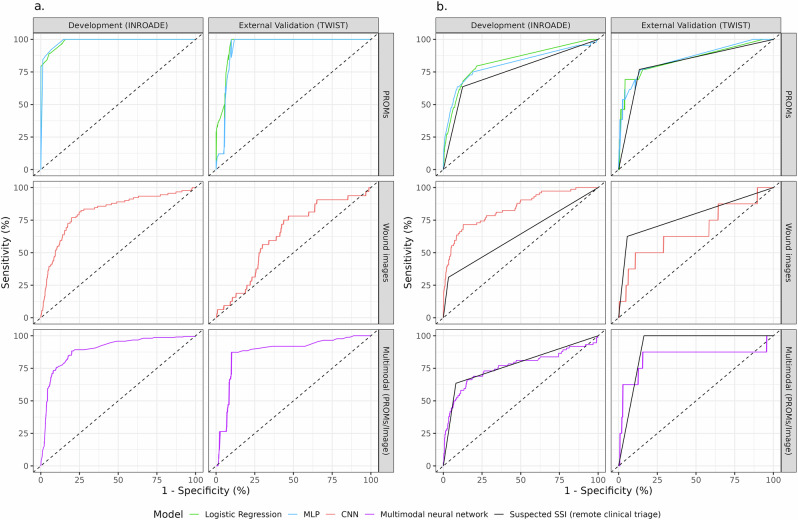
Diagnostic accuracy of different approaches of assessment of patient-reported symptoms and wound images for the prediction of suspected and confirmed diagnosis of SSI within 48 h. Depicts ROC (receiver operating characteristic) curves for each neural network model developed and externally validated. This is shown for (**a**) Suspected SSI on remote clinical triage, (**b**) confirmed SSI on in-person assessment, benchmarked against suspected SSI on remote clinical triage.

Clinician suspicion of SSI based on remote review could be predicted with almost complete accuracy whether an multilayer perceptron (MLP) model (0.988, 95% CI: 0.983–0.993) or logistic regression approach (0.984, 95% CI: 0.978–0.991) was used within INROADE, with equivalent model performance on external validation (Fig. 1, Table 2). While prediction of confirmed SSI within 48 h was significantly lower for both modelling approaches, discrimination remained excellent. Notably, these approaches both demonstrated equivalence to clinician performance at “ruling out” PROMs with no suspicion of SSI on remote review.

**Table 2:** Diagnostic accuracy (95% CI) of different approaches of assessment of patient-reported symptoms and wound images for the diagnosis of SSI within 48 h across the development (INROADE) and external validation (TWIST) datasets

			Clinical classification		SSI within 48 h
Dataset	Approach	Development (INROADE)	External validation (TWIST)	Development (INROADE)	External validation (TWIST)
PROMs	Logistic Regression^a^	0.984 (0.978–0.991)	0.957 (0.938–0.976)	0.830 (0.765–0.895)	0.848 (0.722–0.974)
	MLP	0.988 (0.983–0.993)	0.939 (0.915–0.963)	0.811 (0.732–0.890)	0.854 (0.738–0.971)
	Clinician assessment	–	–	0.756 (0.683–0.828)	0.818 (0.697–0.938)
Wound images	CNN	0.817 (0.768–0.867)	0.636 (0.543–0.729)	0.841 (0.790–0.892)	0.671 (0.437–0.904)
	Clinician assessment	–	–	0.639 (0.586–0.692)	0.784 (0.605–0.964)
Multimodal (PROMs / wound images)	Multimodal neural network	0.893 (0.870–0.917)	0.875 (0.831–0.918)	0.762 (0.690–0.835)	0.834 (0.609–1.000)
	Clinician assessment	–	–	0.777 (0.721–0.832)	0.918 (0.902–0.935)

*CNN* Convolutional neural network, *MLP* Multilayer perceptron (MLP), *PROMs* Patient-reported outcome measures, *SSI* Surgical-site infection.

^a^Models are reported in Supplementary Tables 2, 3.

There were 2615 images collected across the clinical studies, with similar but low event rates between the development (suspected SSI rate on remote triage = 4.3% [*n* = 91/2125], confirmed SSI rate within 48 h = 3.5% [*n* = 74/2125]) and external validation datasets (suspected SSI rate on remote triage = 6.5% [*n* = 32/490], confirmed SSI rate within 48 h = 1.6% [*n* = 8/490]).

**Fig. 2:**
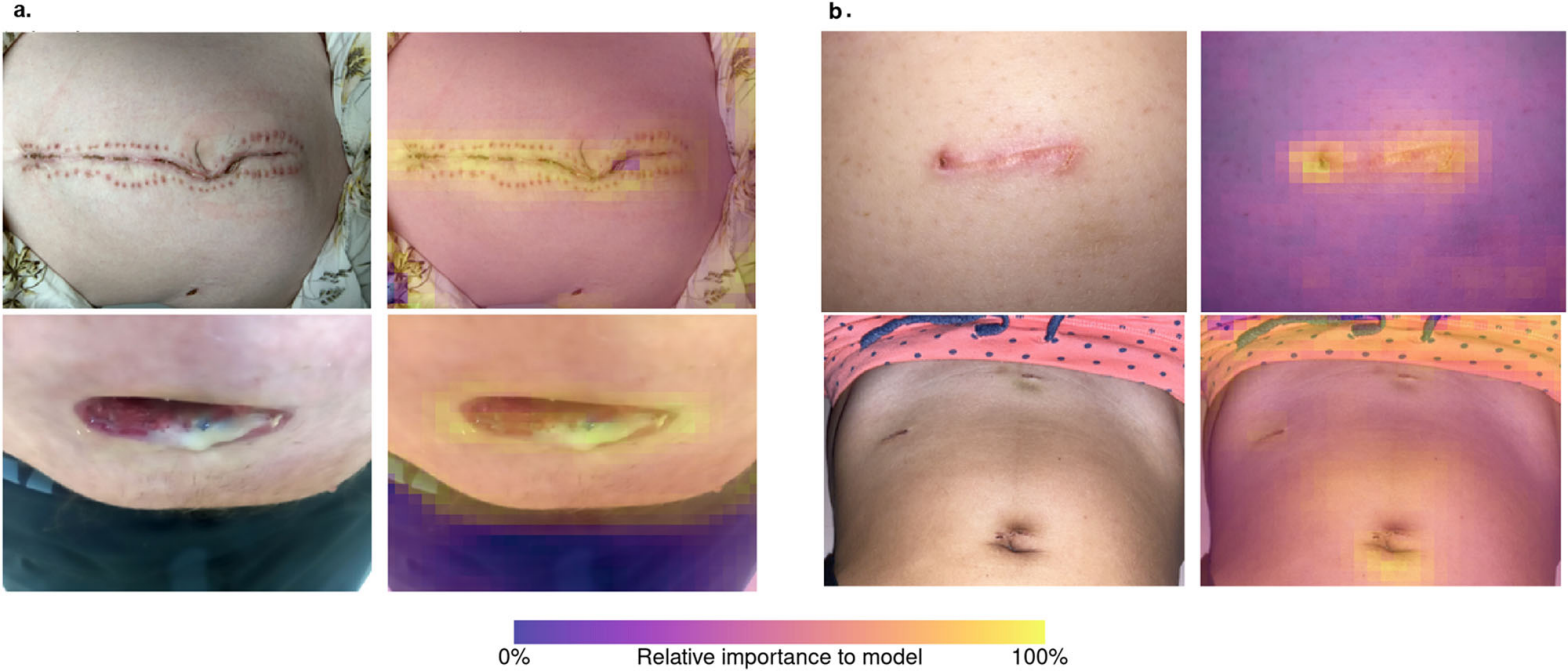
Class activation heatmaps. Depicts the original wound images and images enhanced with class activation heatmaps from the convolutional network model. This is shown for (**a**) images with confirmed SSI within 48 h, (**b**) images with no suspicion or diagnosis of SSI within 48 h.

Using these data, independent convolutional neural network (CNN) models were developed for both outcomes of interest. While excellent model performance was observed to predict the suspected SSI on remote triage within the development data, this reduced on external validation for both outcomes of interest (Fig. 1; Table 2). Class activation heatmaps were used to explore model attention and demonstrated enhancement for clinically important features in the visual assessment of SSI (Fig. 2).

Multimodal neural networks were constructed, incorporating the respective MLP and CNN models. Excellent model performance was observed to predict suspected SSI based on remote review of both PROMs and wound images within the development data, and this remained unchanged on external validation (Fig. 1; Table 2). Furthermore, the multimodal neural network model to predict confirmed SSI within 48 h remained comparable to clinician triage across both datasets, with an excellent performance on external validation.

**Fig. 3:**
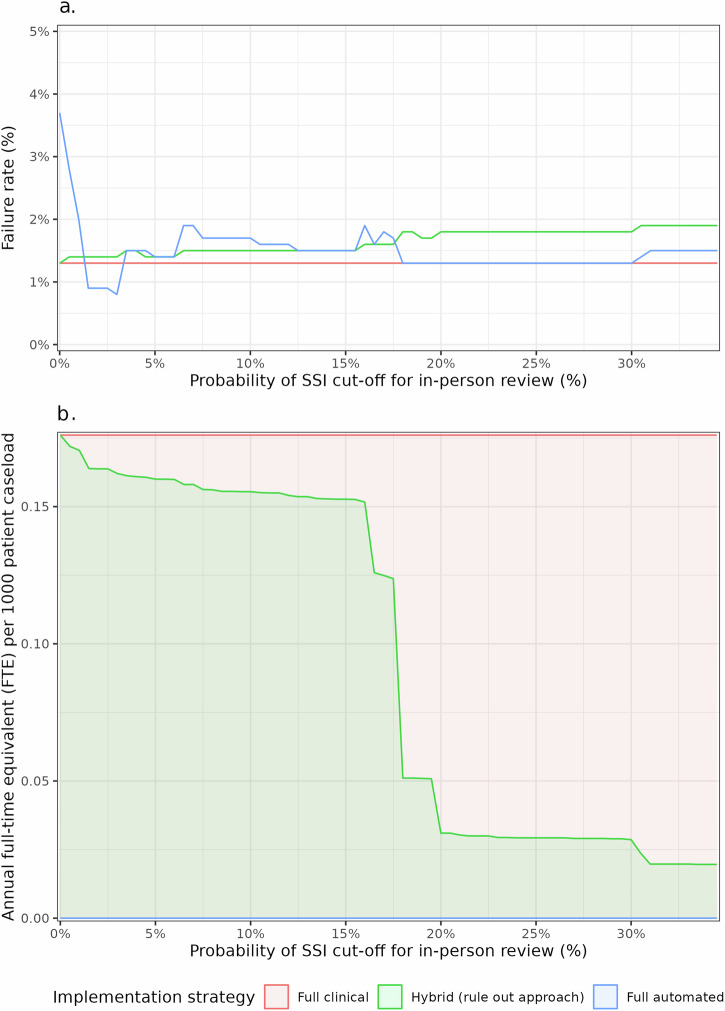
Sensitivity analysis of the simulated implementation of automated assessment strategies in practice, by thresholds for the probability of SSI (%) according to the multimodal model. Depicts the sensitivity analysis of the simulated implementation of automated assessment strategies in practice, by thresholds for the probability of SSI (%) according to the multimodal model. This is shown for (**a**) the failure rate (1- negative predictive value [NPV]), (**b**) the burden on healthcare staff (annual full-time equivalent (FTE) per 1000 patient caseload for clinician triage).

The baseline pathway (full clinical assessment) used in the clinical studies (Supplementary Fig. 2a) was compared to the use of automated assessment to rule-out “low risk” patient responses prior to subsequent clinical assessment (Supplementary Fig. 2b) or to full automation of assessment (Supplementary Fig. 2c). This was simulated in a sensitivity analysis across a spectrum of cut-off values for the thresholds for the probability of SSI according to the multimodal model (Fig. 3).

An exemplar threshold of ≤20% for the predicted probability of SSI was explored (Table 3). Both approaches for automated assessment maintained an excellent discrimination for confirmed SSI within 48 h across the data with a low failure rate, and this was equivalent to clinician review. Use of automated assessment to screen out “low-risk” responses prior to clinical review was associated with a significant reduction in staff-hours to triage responses within a hybrid assessment pathway (9.1 h (−82.4%)). This corresponded to a reduction in the annual full-time equivalent (FTE) per 1000 patient case-load from 0.176 to 0.031.

**Table 3:** Examples of the impact of implementation strategies of automated triage within a remote postoperative monitoring pathway, in comparison to full clinical assessment

		Implementation strategy within remote postoperative monitoring pathway		
		Full clinical assessment	Hybrid assessment (rule out)	Full automated assessment
Performance	Cut-off probability for model	–	0.2	0.2
	Area under the curve (95% CI)	0.789 (0.728–0.850)	0.744 (0.678–0.809)	0.773 (0.712–0.834)
	Sensitivity (95% CI)	0.684 (0.564–0.805)	0.544 (0.415–0.673)	0.702 (0.583–0.821)
	Specificity (95% CI)	0.894 (0.878–0.909)	0.944 (0.932–0.955)	0.844 (0.826–0.863)
	Positive predictive value (95% CI)	0.198 (0.142–0.254)	0.270 (0.188–0.351)	0.147 (0.105–0.189)
	Negative predictive value (95% CI)	0.987 (0.981–0.993)	0.982 (0.975–0.989)	0.987 (0.980–0.993)
	Failure rate (95% CI)	1.3% (0.7–1.9)	1.8% (1.1–2.5)	1.3% (0.7–2.0)
Burden to health service	Responses requiring clinical triage	1545 (100.0%)	272 (17.6%)	0 (0.0%)
	Staff-hours to triage (% reduction)	25.8 (0.0%)	9.1 (−82.4%)	0.0 (−100.0%)
	Annual FTE / 1000 patient case-load (% reduction)	8.3% (−0.0%)	0.031 (−82.4%)	0.000 (−100.0%)
Clinical outcome	No in-person review (low risk)	1348 (87.2%)	1430 (92.6%)	1273 (82.4%)
	- No SSI within 48	1330 (98.7%)	1404 (98.2%)	1256 (98.7%)
	- SSI within 48	18 (1.3%)	26 (1.8%)	17 (1.3%)
	In-person review (moderate-high risk)	197 (12.8%)	115 (7.4%)	272 (17.6%)
	- No SSI within 48	158 (80.2%)	84 (73.0%)	232 (85.3%)
	- SSI within 48	39 (19.8%)	31 (27.0%)	40 (14.7%)

*FTE* full-time equivalent, *SSI* surgical-site infection.

Furthermore, there was also significantly higher specificity for SSI diagnosed within 48 h compared to full clinical assessment (0.944 [95% CI: 0.932–0.955] vs 0.894 [95% CI: 0.878–0.909]), leading to a significant reduction in the recommendation for in-person review (115 (7.4%) vs 197 (12.8%), *p* < 0.001). In comparison, full automated assessment removed the burden on staff to review responses, and provided the most sensitive approach to identify SSI diagnosed within 48 h. However, due to the higher rates of recommendation for in-person review (272 (17.6%) vs 197 (12.8%), *p* < 0.001) this still poses a potentially avoidable burden on healthcare services to review.

## Discussion

Surgical demand and the burden posed by SSI is expected to continue to grow with efforts to provide universal healthcare coverage and to address the post-pandemic elective surgical backlog^23,24^. This creates opportunities for the proposed intervention to reduce the pressure on health services, yet also an incentive to incorporate solutions to minimise its own burden to remain feasible to deliver. These analyses were based on patient-generated data from the largest prospective interventional studies on remote postoperative wound monitoring. Neural networks were used to derive novel approaches for the automated assessment of both PROMs and surgical wound images to identify wounds at “low risk” of SSI. The final multimodal model performance was equivalent to clinician review for the identification of wounds with SSI within 48 h confirmed on in-person clinical review, and model performance remained consistently excellent on external validation. When implementation within remote postoperative wound monitoring pathways was simulated, automated exclusion of “low risk” wounds was estimated to reduce the staff-time required to deliver by over 80%, while maintaining a low failure rate when compared to a full clinician-led pathway.

With any modelling approach, it is essential that the features being identified are of clinical importance. Of the patient-reported symptoms related to SSI, there was significant heterogeneity in those reported (Supplementary Fig. 1). All symptoms except wound oedema were significantly associated with confirmed SSI within 48 h (Supplementary Table 2), although only wound erythema and discharge were independently associated. The CDC criteria^20^ assumes that all symptoms of inflammation are of equal clinical significance. These represent the most clinically obvious and late-onset symptoms of infection, which does not preclude the other symptoms providing an equal or potentially more significant diagnostic or prognostic role (even if patients may be less reliably able to identify these). In rare cases (Supplementary Fig. 1), patients did not report any clinical symptoms of SSI yet were still diagnosed with an SSI within 48 h. This indicates that either there can be rapid change in the presentation of symptoms in some cases, or that a minority of patients may find it difficult to reliably determine symptoms of SSI. The explainability of the CNN models was also explored using class activation heatmaps to ensure predictions were aligned with the pathognomonic features of SSI (Fig. 2). Within this context the class activation heatmaps demonstrated correlation with the wound itself, but more specifically wounds that had been highlighted as having evidence of SSI by clinical staff, such as erythema or purulent fluid being present. This ensures that the CNN predictions are transparent and explainable, and so aids in bridging the gap in trust between machine learning, and healthcare staff and patients^25–27^.

These methods of automated assessment of patient-generated were developed to identify wounds which could be confidently evaluated to be at “low risk” of SSI (“rule out” approaches). While diagnostic accuracy based on PROMs was excellent for both outcomes of interest, and remained so on external validation (Table 2). Furthermore, the performance of the multimodal models was similar to the DeepWound CNN model (AUC: 0.84)^28^, despite DeepWound being trained on image data with a higher event rate of SSI (26.6% vs 3.5% in INROADE). While only moderate discrimination observed for the CNN models on external validation (Table 2), it should be noted that this has not yet been performed for the DeepWound CNN model. Overall, clinical triage remained typically the most accurate method to assess wound images and identify those which would receive an SSI diagnosis within 48 h (Table 2). However, an equivalent diagnostic accuracy was achievable with the multimodal NN, demonstrating the capability to approximate clinician assessment. This was confirmed in practice when both strategies for the implementation of the multimodal neural network model as part of automated assessment pathways maintained diagnostic accuracy equivalent to the baseline pathway (Supplementary Fig. 2; Table 3). These pathways have a distinct advantage in allowing a substantial reduction in the burden of staff to deliver (Table 3; Fig. 3). However, only the hybrid approach also reduced the number of patients recommended for in-person review. This suggests that while these models can accurately “rule out” responses without clear evidence of SSI, clinicians currently remain superior at reviewing more complex cases.

This work presents the most comprehensive analysis to date of methods for automated remote assessment of surgical wounds, with several notable strengths to the approach. Firstly, the combination of the TWIST and INROADE studies represents one of the largest repository of surgical wound images for research, and the only known dataset of surgical wounds annotated by both clinicians and patients for the presence of SSI. Together with well-established deep learning techniques, this allowed the first known multimodal neural network model to provide a unified approach for predicting of surgical-site infection. This incorporated the first known application of class activation heatmaps to surgical wounds published to date, enhancing transparency and interpretation of predictions made. Furthermore, this analysis also incorporated evaluation of not just model performance on external validation, but also considered the healthcare impact within clinical pathways. This was guided by stakeholder input^11^, and allowed quantification of the potential impact on the burden to deliver as well as the diagnostic accuracy of remote triage.

However, there were also several important limitations. Firstly, there was an overall low event rate within the development dataset, with only 3.7% (*n* = 57/1540) of responses submitted within 48 h of an SSI diagnosis. This is partially attributable to patient adherence, but also the lack of direct clinical validation of patient responses in comparison to the gold-standard of in-person assessment within the studies. When patients received a clinical recommendation, it was up to them to decide if and where to attend for further clinical assessment. This is reflective of how the intervention would function in practice. However, unless in-person review was conducted swiftly, there may be evolution in the surgical wound in the interim, and so a discrepancy with the patient-submitted data. While this low event rate limits the statistical power of modelling approaches for the diagnosis of SSI, the intended use case for the models developed in the analysis was for the “rule out” of wounds at a low-risk of infection to reduce the burden to deliver remote postoperative wound surveillance. Secondly, the hospitals involved in the clinical studies serve a predominantly White ethnic population, and so few patients of other ethnic backgrounds were able to be enrolled (Table 1). It is well established that there is an underrepresentation of darker skin tones within medical learning materials and image repositories^29,30^. Both clinicians and deep learning models can perform poorly at identification of the visual components of the SSI diagnostic criteria which can present differently in these patients^20^. Further training and validation of remote wound assessment across a spectrum of skin tones is essential when implementing in routine care, particularly in settings with more ethnic diversity. Thirdly, due to the nature of patient-generated images with smartphone cameras, these can be heterogenous in resolution and visual elements. CNN models rely on high quality and standardised images, and/or a large volume of images to have robust performance. Therefore, future work to expand the image repository used for model training would be expected to improve performance further for detection of SSI, as well as sophisticated augmentation strategies or transfer learning from datasets with more diverse skin tones. Finally, these automated assessments only utilised information available at a single point in time. Further improvement in model performance may be achieved through the use of models to account for the evolving nature of the risk of SSI in the postoperative period^31–33^. This would have the potential to provide new insights into the diagnosis of SSI at a subclinical stage, and even prediction of wounds at high-risk to allow preventative interventions.

There has been growing recognition in recent years from governments and healthcare organisations that digital transformation is not just desirable, but essential to healthcare delivery in the future^4^. However, decision assistance in the form of algorithms or thresholds for alerts will be vital for the anticipated benefits to be realised in practice, particularly as the amount and complexity of data collected on patients expands. In this regard, this work provides a foundation which can be emulated in the context of other digital surveillance services seeking to formally incorporate algorithm-based decision support or triage. Nevertheless, despite the clear evidence for clinical utility for these models within a remote monitoring pathways, this does not gurantee clinical adoption^34^. There are several issues that need to be addressed before automated assessment can be more widely used within clinical pathways. Firstly, the use of automated assessment within a remote postoperative surveillance service would involve at least partial replacement of clinical decision-making. Therefore, these models would be classified as a medical device and would require regulatory approval prior to formal clinical use. Secondly, stakeholders must also have confidence in the results of automated assessment to continue to engage^35,36^. Key drivers of engagement in remote monitoring for many patients are “speaking to an expert” and more personalised interactions with healthcare staff^11,19^, which may be affected by the partial automation of triage. Furthermore, there are wider apprehensions regarding the safety and trustworthiness within the healthcare context^27^, which may reduce engagement and concordance with recommendations among all stakeholders. Therefore, further work to explore barriers and effective solutions for the use of automated approaches in remote triage would be warranted. This may include clinical validation studies to allow direct correlation of in-person examination findings with patient-generated data^11^; improving explainability of the rationale behind model decision-making, such as real-time class activation heatmaps; and to evaluate automated assessment using patient-generated data within independent studies, particularly among populations with more diverse skin tones when using image-based data as in this context^37^.

Overall, this study has demonstrated that automated assessment can be successfully deployed within remote postoperative wound surveillance pathways to reduce the burden on healthcare staff to deliver without compromising care, thus allowing resources to be appropriately directed to those at greatest risk of SSI. As digital transformation of healthcare continues, implementation of these methods within care pathways will require engagement of all stakeholders to ensure this can be integrated in a safe, transparent, and acceptable manner.

## Methods

This study reports the derivation and validation of a proof-of-concept deep learning model to allow automated stratification of abdominal surgical wounds according to their risk of SSI. This study was reported according to the “*Transparent reporting of a multivariable prediction model for individual prognosis or diagnosis*” (TRIPOD) statement^38^.

### Data sources

This was an analysis of multimodal patient-generated data from two prospective interventional studies conducted on the use of digital remote postoperative wound monitoring: “*Tracking wound infection with smartphone technology*” (TWIST)”^18^ and “*ImplementatioN of Remote Surgical wOund Assessment during the coviD-19 pandEmic*” (INROADE)^19^. These included eligible adult patients (age ≥ 18 years) undergoing gastrointestinal surgery across two participating hospitals within a single UK health board (NHS Lothian) enrolled between July 2016 to March 2020 (TWIST) and July 2021 to April 2022 (INROADE). Research Ethics Committee (REC) approval was received for both TWIST (South-East Scotland: 16/SS/0072), and INROADE (West of Scotland: 21/WS/0046)), and all patients provided informed consent to participate. This encompassed use of data for secondary analysis.

The methods and primary findings, and the principal components of the digital remote postoperative wound monitoring intervention have been previously described in the original studies^18,19^. The intervention was consistent across both studies, with patients given access to the online platform throughout the early postoperative period (postoperative day 1–30). Patients were able to submit an image of their surgical wound(s), and a series of patient-reported outcomes (PROMs) related to surgical-site infection (Supplementary Table 3). These PROMs were simple questions used to establish the presence or absence of signs and symptoms indicative of SSI according to the patient, with branching questions added in the INROADE study to also quantify the perceived changes over time of each symptom (“*new onset*”, or “*worse*”, “*same*”, or “*better*” compared to the last submission). These patients were aware that data they submitted may be used for the purposes of machine learning but that this would have no impact on the clinical recommendations received within the studies; therefore, no blinding to outcomes or other predictors was deemed necessary.

### Outcomes of interest

Prediction of two outcomes of interest were explored in independent modelling frameworks. Firstly, suspected SSI on remote clinical triage – yes (moderate- or high risk of SSI) or no (low risk of SSI). This was intended to reduce the burden of clinical triage of patient responses. Each individual submission was reviewed by a qualified clinician trained to recognise surgical-site infection. The evidence of SSI on patient-reported symptoms and wound images was classified independently and then overall as either: (1) that there was no clear evidence of SSI present (low risk), but with recommendation to attend healthcare services or submit a further form if ongoing concerns; (2) possible evidence of SSI (moderate-risk), with recommendation to attend community healthcare services for clinical review; or (3) probable evidence of SSI (high risk), with recommendation to attend emergency services for clinical review. Secondly, the confirmed diagnosis of SSI on in-person clinician review within 48 h of the submission of the response - yes or no. This sought to determine whether evidence of SSI can be identified de novo, benchmarked against clinical triage of patient responses. Diagnosis of SSI was recorded according to the Centers for Disease Control and Prevention (CDC) definition^20^ within the 30-day postoperative period, and was determined using a combination of: (1) the telephone follow-up of the patient; (2) electronic patient record review; and (3) review of wound logs which documented any wound reviews in the community (returnable in a pre-paid envelope).

### Statistical analysis

All statistical analyses were performed in R Studio version 4.1.1 (R Foundation for Statistical Computing, Vienna, Austria), with packages including tidyverse, keras, finalfit, and predictr^39^. Numerical data were summarized as mean (standard deviation) or median (interquartile range) based on visual and statistical evaluation for normality, with appropriate tests for parametric or non-parametric data performed. Categorical data were cross-tabulated, and tested using χ2 or Fisher’s exact tests.

### Neural network modelling

**Fig. 4:**
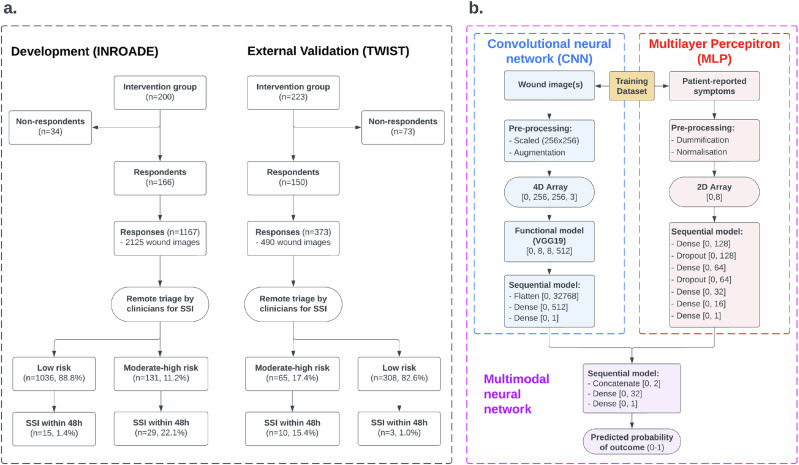
Data flowcharts. Depicts the flow of data throughout the analysis. This is shown for (**a**) all patients in both the TWIST and INROADE studies, (**b**)all components of the multimodal neural network framework.

Model development was conducted separately for patient-reported symptoms and wound images within the INROADE dataset using neural network frameworks (Fig. 4a). These data were randomly split into development (training) and internal validation (testing) datasets in a 4:1 ratio, with repeat responses clustered by patient to ensure no data leakage and stratified by outcome to achieve balanced event rates between datasets. The trained models output a probability of the occurrence of the outcome of interest between 0 and 1 (binary classification). All models were subsequently externally validated within the TWIST dataset. Model performance was compared using the area under the receiver operating characteristic curve (AUC) and prognostic accuracy summary statistics (sensitivity, specificity, positive predictive value [PPV], and negative predictive value [NPV]). An AUC of 0.5–0.59 was considered to be “poor”, 0.6–0.69 was considered to be “moderate”, 0.7–0.79 “good” and ≥0.8 “excellent” model discrimination^40^.

Due to the multimodal nature of the patient response data, three different neural network modelling approaches were explored and compared (Fig. 4b). Firstly, MLP models were developed to predict outcome based on patient-reported symptoms^41^. All PROMs data were categorical, and so were pre-processed using one-hot encoding (dummification) to allow efficient modelling and reduce model bias. Subsequently, these data were normalised to have a mean of zero (centred) and standard deviation of one (scaled). Missing data due to partial completion of PROMs were assumed to have the absence of the respective symptoms given this would be the assumption in clinical practice. These training data were supplied to the sequential MLP model as a 2 dimensional array (0, 8). The performance of this MLP modelling approach was compared against multivariable logistic regression utilising the same input variables. Secondly, CNN models were developed to predict outcome using wound images. These are a form of deep learning which have proven adept at classifying images based on the presence or absence of particular features^13^. Each image submitted by patients across both studies were manually reviewed, and those without a visible wound were discarded. Image data pre-processing was conducted according to standard practice, with all images in colour and scaled to a standardised 256 × 256 pixels^13^. Augmentation was then applied to training data in order to further expand the image dataset and so improve the robustness and generalisability of the subsequent model^13^. This included transformations such as translation, rotation, scaling, and flipping, and avoided shape deformation to preserve wound characteristics. These training data were supplied to the CNN model as a 4 dimensional array (0, 256, 256, 3) which utilised transfer learning from an open-access image classification model to exploit the knowledge gained^42^. In pilot work, we compared VGG19, VGG16, InceptionV3, and ResNet50. VGG19 had the best discrimination with a slightly higher computational cost. This strategy improves generalization as it exploits features extracted in more general settings and reduces chances of overfitting^42^. The layers of the VGG19 model were unfrozen at the 3^rd^ pooling stage to allow fine-tuning. The CNN models were fine tuned for 30 epochs (optimised using root mean square propagation in batch sizes of 10) with early stopping based on training loss (binary cross-entropy) after every ten steps and a patience of ten. Class activation heatmaps were also derived from the images processed by the algorithm^13^. This allowed clinical confirmation that the wound features being identified by the machine learning algorithm were consistent with the known pathognomonic features of SSI. Finally, late fusion was used to combine prior models to form a multimodal neural network using the multimodal data. This allowed incorporation of patient-reported outcomes and wound images within the same predictive framework, and allowed a single prediction based on all data contained in a single patient response to be generated. This approach has been demonstrated to have superior performance to independent models within other contexts^43,44^.

### Simulation of implementation strategies

The proposed use-case for these approaches for automated assessment of online responses was to reduce the burden of clinical triage (by allowing online responses without evidence of SSI to be automatically classified as such). Therefore, the healthcare impact of implementation of automated assessment within the remote postoperative wound monitoring care pathway was explored under different simulated scenarios. The baseline scenario involving full clinical assessment in TWIST and INROADE was established using the sensitivity and specificity for confirmed SSI diagnosis within 48 h (Supplementary Fig. 2a). Subsequently, two scenarios were simulated based on integration of multimodal neural network assessment: “*Hybrid assessment*” involving partial automation to rule-out low risk wounds prior to clinical review (Supplementary Fig. 2b), and fully automated assessment (Supplementary Fig. 2c). For responses where no image was submitted, the prediction from the MLP was used. For responses with multiple images submitted at once, the highest predicted risk was used.

Outcomes assessed across all scenarios included the number of responses reviewed by staff, the overall diagnostic accuracy, the failure rate (the proportion of patients stratified to the low risk group who are then diagnosed with SSI within 48 h [1 – NPV]), and the number of recommended in-person reviews. For the purposes of each scenario, clinical assessment of each response was estimated to take 2 min, whereas automated assessment was assumed to be real-time (0 min). The staff-time required to deliver was standardised as the annual FTE per 1000 patient caseload for clinician triage. This was calculated assuming that full time was 37.5 h per week^45^ (1950 h over 52 weeks per year). This was then scaled according to the number of patients and duration of the INROADE study due to this featuring the more intensive follow-up. Finally, a sensitivity analysis was conducted to vary the threshold for the probability of SSI cut-off for in-person review, and explore effect on the staff-time to deliver and the failure rate.

## Supplementary information

Supplementary Information

## Data Availability

Sharing of patient generated data collected as part of this research is restricted to instances with data access agreements with clear terms which protect confidentiality and intellectual property. However, in line with transparency and reproducibility in scientific research, the code for results presented and deidentified participant data are available on reasonable request from the corresponding author.

## Code availability

The code generated and used during the current study are available from the corresponding author on reasonable request.
