# Supplementary material for: Multimodal machine learning to predict surgical site infection with healthcare workload impact assessment
Source: NPJ Digit Med. 2025 Feb 23;8:121. doi: 10.1038/s41746-024-01419-8 (PMC11847912; doi:10.1038/s41746-024-01419-8)
Supplement: Supplementary file 1 — Supplementary Information [file 41746_2024_1419_MOESM1_ESM.pdf]

Supplement

Supplementary Figure 1: Patient-reported symptoms of SSI according to the clinical risk classification and the rate of SSI diagnosis within 48 hours.

Depicts the patient-reported symptoms of SSI according to the clinical risk classification and the rate of SSI diagnosis within 48 hours. This is shown for (a). the rate of SSI diagnosis within 48 hours of response observed per group; (b) patient-reported symptoms of SSI observed per group

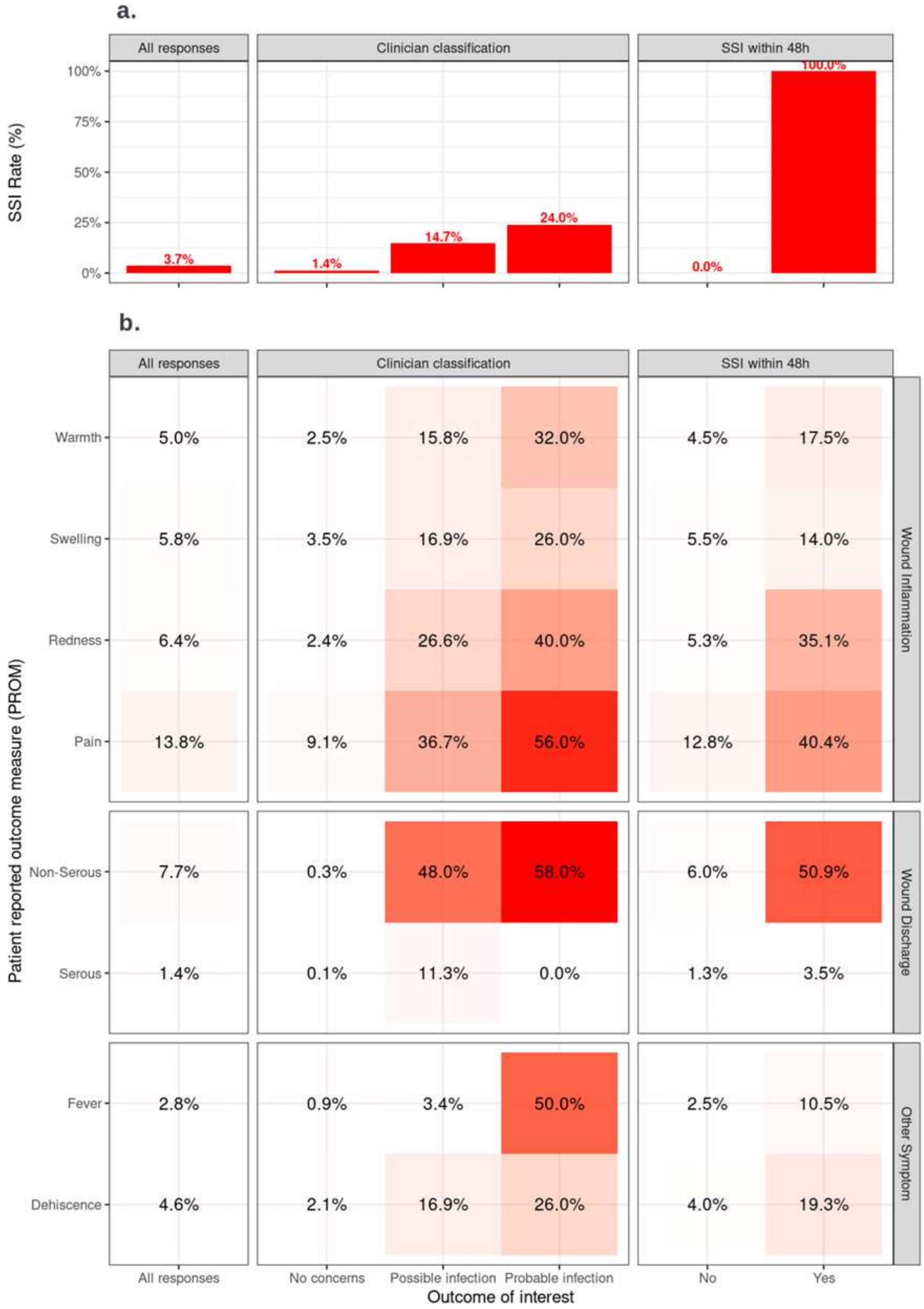

Supplementary Figure 2: Implementation strategies for automated wound assessment for SSI

Depicts the strategies explored for implementation of automated wound assessment for SSI within the proposed remote postoperative wound monitoring pathway. This is shown for (a). the baseline scenario involving full clinical assessment; (b) hybrid integration of multimodal neural network assessment, involving partial automation to rule-out low risk wounds prior to clinical review; and (c). fully automated assessment using a multimodal neural network.

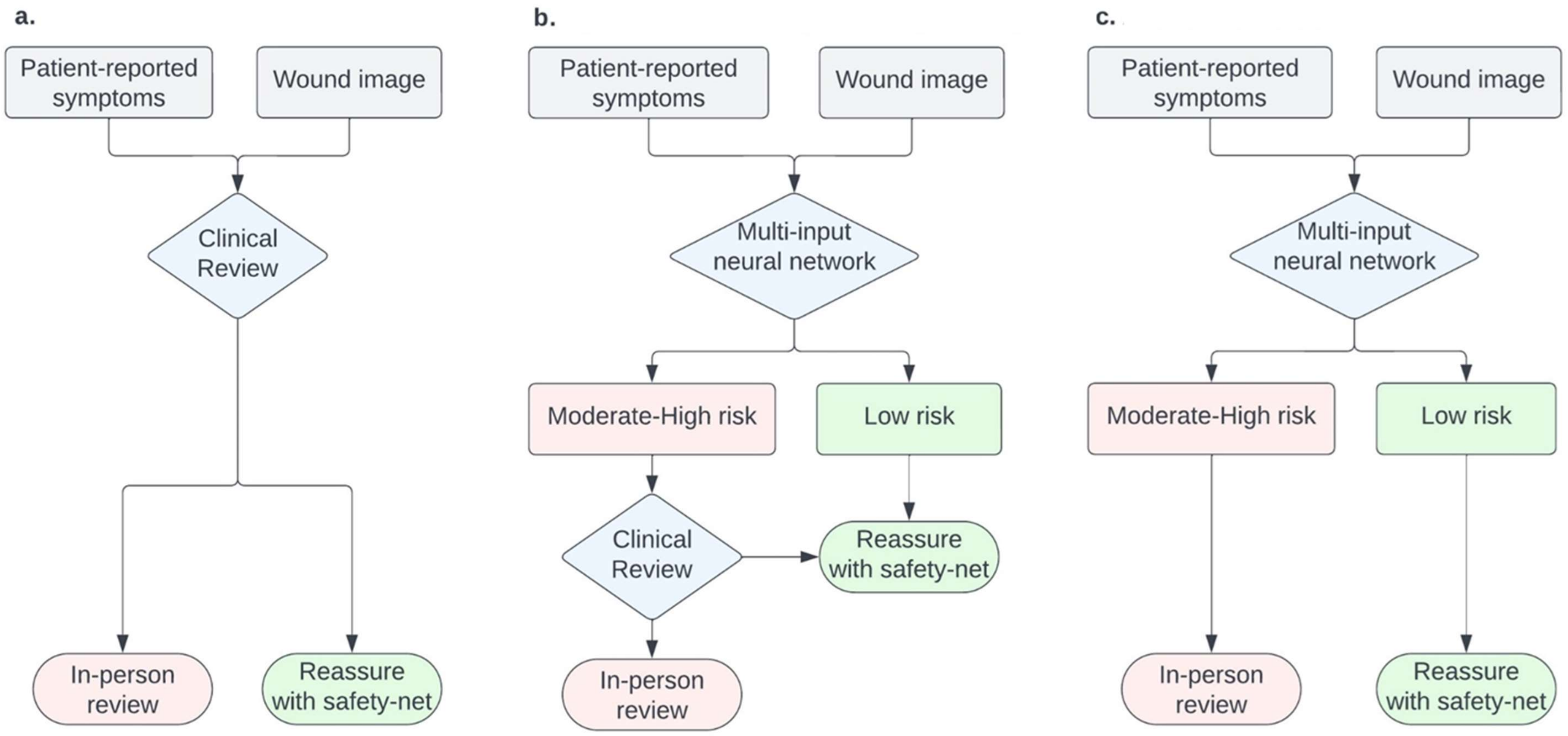

Supplementary Table 1: Multivariable logistic regression using patient-reported symptoms to predict the suspected SSI on remote clinical triage

		Suspected SSI (remote clinical triage)			
		**Concern (n=168)**	**No concerns (n=999)**	**Univariable OR (95% CI)**	**Multivariable OR (95% CI)**
Warmth	No	144 (12.9)	969 (87.1)	-	-
	Yes	24 (44.4)	30 (55.6)	5.38 (3.04-9.45, p<0.001)	1.47 (0.42-4.37, p=0.515)
Swelling	No	145 (13.1)	958 (86.9)	-	-
	Yes	23 (35.9)	41 (64.1)	3.71 (2.13-6.31, p<0.001)	2.46 (0.90-6.16, p=0.065)
Redness	No	130 (11.8)	969 (88.2)	-	-
	Yes	38 (55.9)	30 (44.1)	9.44 (5.67-15.86, p<0.001)	9.09 (3.40-23.36, p<0.001)
Pain	No	101 (10.2)	885 (89.8)	-	-
	Yes	67 (37.0)	114 (63.0)	5.15 (3.57-7.42, p<0.001)	7.27 (3.29-16.14, p<0.001)
Wound discharge	None	49 (4.7)	999 (95.3)	-	-
	Haemoserous	42 (100.0)	0 (0.0)	Inf (0-Inf)*	Inf (0-Inf)*
	Purulent	77 (100.0)	0 (0.0)	Inf (0-Inf)*	Inf (0-Inf)*
Dehiscence	No	135 (12.1)	981 (87.9)	-	-
	Yes	33 (64.7)	18 (35.3)	13.32 (7.38-24.78, p<0.001)	1.11 (0.06-6.51, p=0.924)
Fever	No	148 (12.9)	999 (87.1)	-	-
	Yes	20 (100.0)	0 (0.0)	Inf (0-Inf)*	Inf (0-Inf)*

* No continuity correction applied in the presence of a 0 event. Number in dataframe = 1167, Number in model = 1167, Missing = 0, AIC = 254.9, C-statistic = 0.984, H&L = Chi-sq(8) 0.01 (p=1.000)

Supplementary Table 2: Multivariable logistic regression using patient-reported symptoms to predict confirmed SSI within 48h on in-person clinical assessment

		Confirmed SSI within 48h (in-person clinical assessment)			
		Yes (n=46)	No (n=1123)	Univariable OR (95% CI)	Multivariable OR (95% CI)
Warmth	No	38 (3.4)	1075 (96.6)	-	-
	Yes	6 (11.1)	48 (88.9)	3.54 (1.29-8.21, p=0.006)	0.76 (0.21-2.39, p=0.658)
Swelling	No	39 (3.5)	1064 (96.5)	-	-
	Yes	5 (7.8)	59 (92.2)	2.31 (0.78-5.59, p=0.089)	0.57 (0.14-1.96, p=0.407)
Redness	No	30 (2.7)	1069 (97.3)	-	-
	Yes	14 (20.6)	54 (79.4)	9.24 (4.52-18.17, p<0.001)	5.67 (2.27-13.61, p<0.001)
Pain	No	28 (2.8)	958 (97.2)	-	-
	Yes	16 (8.8)	165 (91.2)	3.32 (1.72-6.20, p<0.001)	1.52 (0.67-3.28, p=0.299)
Wound discharge	None	20 (1.9)	1028 (98.1)	-	-
	Haemoserous	7 (16.7)	35 (83.3)	10.28 (3.83-24.96, p<0.001)	6.29 (2.02-17.42, p=0.001)
	Purulent	17 (22.1)	60 (77.9)	14.56 (7.19-29.27, p<0.001)	11.94 (5.44-25.74, p<0.001)
Dehiscence	No	37 (3.3)	1079 (96.7)	-	-
	Yes	7 (13.7)	44 (86.3)	4.64 (1.81-10.43, p<0.001)	0.77 (0.24-2.21, p=0.642)
Fever	No	41 (3.6)	1106 (96.4)	-	-
	Yes	3 (15.0)	17 (85.0)	4.76 (1.08-14.88, p=0.016)	2.38 (0.45-9.12, p=0.246)

* Number in dataframe = 1167, Number in model = 1167, Missing = 0, AIC = 317, C-statistic = 0.83, H&L = Chi-sq(8) 7.42 (p=0.492)

Supplementary Table 3: Smartphone-delivered wound assessment tool incorporating patient-reported outcome measures and wound image submission.

Question	Options
1. Has the wound been painful to touch	No, Yes
2. Is there liquid coming from the wound site	No, Yes
3. Is there redness spreading away from the wound?	No, Yes
4. Has the area around the wound become swollen?	No, Yes
5. Was the area around the wound warmer than the surrounding skin?	No, Yes
6. Have the edges of any part of the wound separated/gaped open of their own accord?	No, Yes
7. Have you had, or felt like you have had, a raised temperature or fever? (fever > 38 °C)	No, Yes
8. Have you been diagnosed with a wound infection since surgery?	No, Yes
9. Please upload a photograph of your wound.	-
